# Digital eyes on diabetes: a systematic review of attitudes toward telemedicine-based retinopathy screening

**DOI:** 10.1093/oodh/oqaf032

**Published:** 2025-12-04

**Authors:** Suraj Patil, Judy Jenkins, Jomin George

**Affiliations:** Department of Health Informatics, Swansea University, Singleton Park, Swansea SA2 8PP, Wales, United Kingdom; Department of Health Informatics, Swansea University, Singleton Park, Swansea SA2 8PP, Wales, United Kingdom; School of Nursing and Midwifery, Royal College of Surgeons, 123 St Stephen's Green, Dublin 2, D02 YN77, Ireland

**Keywords:** diabetic retinopathy, telemedicine, screening, meta-ethnography, patient attitudes, healthcare providers, qualitative research

## Abstract

Diabetic Retinopathy (DR) is a leading cause of vision loss among people with Diabetes Mellitus worldwide. Early stages are asymptomatic, making timely screening essential to prevent irreversible damage. Telemedicine offers a promising avenue to improve screening accessibility, especially where specialist services are limited. This study aims to explore the attitudes of patients and healthcare providers towards telemedicine-based screening for Diabetic Retinopathy. Objectives include identifying beliefs, biases and barriers influencing the adoption of teleophthalmology for DR screening. A meta-ethnography was conducted, synthesising qualitative studies from PubMed, Scopus and MEDLINE that utilized interviews, focus groups and document analysis to investigate perceptions of telemedicine in DR screening. Nineteen studies met the inclusion criteria and underwent quality appraisal. Five higher-order themes emerged: lack of knowledge, economic factors, provider challenges, ease of integration and perceived benefits of screening. Patient non-attendance was largely due to low awareness and asymptomatic disease perception, while providers faced training, technical and referral pathway challenges. Telemedicine’s integration requires leadership engagement and clear workflows. Both patient and provider perspectives significantly influence telemedicine adoption for DR screening. Addressing knowledge gaps, financial barriers and provider training, alongside streamlined referral systems, could enhance screening uptake and effectiveness. This synthesis uniquely highlights the complex psychosocial and systemic factors affecting telemedicine-based DR screening acceptance, providing actionable insights for improving screening programmes globally.

## INTRODUCTION

Nearly 422 million people worldwide are affected by Diabetes Mellitus [1], representing nearly 9% of the global population [2]. This condition, characterized by elevated blood glucose, can damage retinal blood vessels, leading to Diabetic Retinopathy and vision impairment [3–5]. Early stages are often asymptomatic, causing gradual fundus deterioration until treatment is less effective [6–8]. Thus, regular dilated fundus screening is vital for early detection [8, 9]. A landmark laser photocoagulation study showed that consistent monitoring over five years significantly reduced visual decline [10]. Telemedicine is crucial in resource-poor areas, as patients travelling over an hour for eye care are less likely to attend screening [11]. Most diabetics see General Physicians who may lack Diabetic Retinopathy expertise [11, 12], yet GPs and Nurses can perform fundus imaging and educate patients [13–15]. Screening at primary care centres improves compliance [16, 17], and telemedicine enhances Ophthalmologist-GP collaboration [18]. Smartphone fundus cameras offer high accuracy [19–21], with 30 minutes’ training improving image quality [22]. However, Ophthalmologists remain uncertain of their telemedicine roles [23]. Patient education and reminders aid attendance [24–26], though findings vary [27], highlighting the need for qualitative insights from patients and providers.

### Research question

What are the attitudes of patients and healthcare providers towards the use of telemedicine for the screening of diabetic retinopathy?

### Aims

This study aims to understand the perspectives of providers and patients towards the use of Telemedicine for the screening of Diabetic Retinopathy.

### Objectives

- To identify the attitudes of patients and providers regarding the screening of Diabetic Retinopathy with the help of Telemedicine.
- To examine the beliefs and potential biases of providers during the adoption of Teleophthalmology for screening of Diabetic Retinopathy.

### Rationale

To increase the screening rates, a strong understanding of the barriers and enablers to screening from both the patient-perspective and provider perspective is required. In high income countries (HICs), barriers often relate to fragmented healthcare systems, cost effective, cost effectiveness concerns and technology integration [41, 54, 58, 70]. By contrast, in low and middle-income countries (LMICs), challenges are more likely tied to work workforce shortages, lack of infrastructure and limited patient awareness [51, 59, 60]. Addressing these context specific barriers can significantly improve uptake and sustainability of teleophthalmology programmes.

### MATERIALS AND METHODS

Several KAP studies across countries assess patients’ awareness and attitudes towards diabetic retinopathy screening and treatment [28–32]. However, KAP studies lack scrutiny of interviewees’ internal beliefs influencing behaviour [33]. For complex phenomena beyond binary responses, qualitative research is preferred, offering deeper understanding regardless of sample size [34]. This study explores disinterest in telemedicine screening via qualitative synthesis, adopting meta-ethnography. Meta-ethnography interprets and translates concepts across studies, grouping ideas into higher-order interpretations [35]. It uncovers psychological reasons behind non-attendance, using critically appraised IDI, FGD, document analysis and observation studies [36–38]. Mixed methods were excluded [36].

### Search strategy

Academic databases including PubMed, Scopus and MEDLINE were searched using terms such as ‘diabetic retinopathy’, ‘screening’ and ‘telemedicine’, combined with ‘focus group’, ‘interview’, ‘attitude^*^’, ‘feeling’, ‘perception’, ‘practice’, ‘satisfaction’ and ‘qualitative^*^’ with Boolean operators AND/OR, and filtered by the PRISMA checklist [39]. The SPIDER tool as depicted in Table 1 below, was preferred over PICO for qualitative research due to indexing issues [38]. Free-text searches were used instead of MeSH terms [40]. Only English-language studies were included; editorials, letters, quantitative studies, proposals and studies on other ocular diseases were excluded. Abstracts were screened and duplicates removed; full texts reviewed via EndNote, retaining only studies meeting strict criteria.

**Table 1:** Qualitative search strategy: SPIDER framework.

SPIDER Tool	Meaning	Search Terms
S	Sample	‘patient^*^’ OR ‘provider^*^’ OR ‘clinician^*^’ OR ‘physician^*^’
PI	Phenomenon of Interest	‘Diabetic retinopathy’ AND ‘screening’
D	Design	‘interview’ OR ‘focus group’
E	Evaluation	‘feeling’ OR ‘perception’ OR ‘attitude’ OR ‘satisfaction’
R	Research Type	‘qualitative^*^’

## RESULTS

A total of 619 articles were identified through the search strategy. Excel records from each academic database were downloaded, merged into a single sheet, retaining only the ‘Authors’ and ‘Title’ columns. An additional ‘Source’ column indicated the database of origin. Articles were sorted by ‘Title’ to place potential duplicates adjacent. A new ‘Duplicate’ column was added, using the formula ‘(A2) = (A1)’ to flag duplicates, which returned ‘True’ where matches occurred. These were filtered out, and the remaining unique records were transferred to a new sheet. An ‘Assessment’ column was introduced to document outcomes of the full-text review. After removing duplicates, 341 articles remained for abstract review. From these, 207 were selected for full-text review, and ultimately, 19 were included in this review. Quality appraisal of the final set of studies was conducted using the CASP (Critical Appraisal Skills Programme) checklist. This systematic process ensured a rigorous and transparent selection of relevant literature.

**Figure 1:**
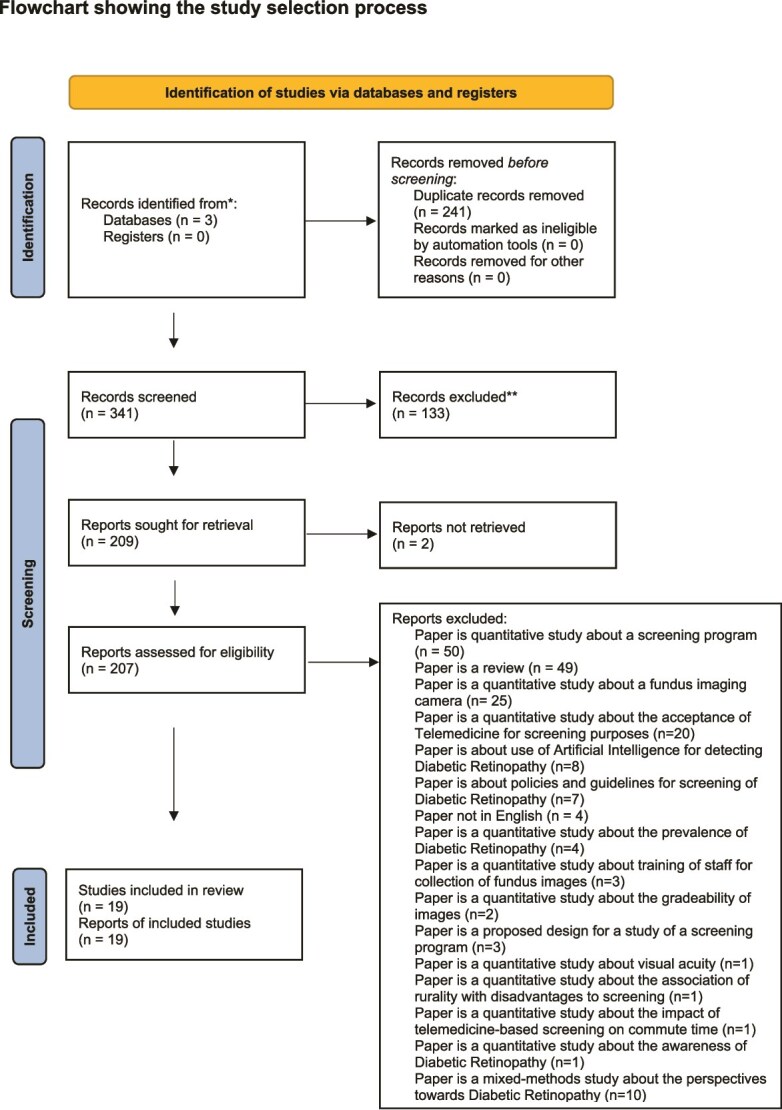
Flowchart showing the study selection process.

As depicted in Fig. 1 above, out of the 19 selected studies, 10 were from the USA [7, 41–49], 2 from India [50, 51], 1 from China [52], 2 from Australia [53, 54], 2 from Canada [55, 56], 1 from Portugal [57], 1 from Germany [58] and 1 spanned four African countries: Ghana, Botswana, Zimbabwe and Tanzania [59]. Most used semi-structured interviews (n = 17) [41–43, 45–50, 52–59], followed by focus groups (n = 4) [7, 43, 44, 55] and document analysis (n = 1) [59]. Data analysis methods included framework fit (n = 10) [41, 45–48, 50, 52, 54, 58, 59], thematic (n = 4) [49, 55–57], grounded theory (n = 2) [42, 53], with some unreported (n = 4) [1, 43, 44, 60]. NVivo was most used (n = 9) [42, 44–46, 49, 50, 52, 53, 60], then Atlas.ti (n = 2) [7, 41], Scrivener (n = 1) [57] and MAXQDA (n = 1) [48].

### Quality of the studies

All included studies had clear aims aligned with the research question and systematically communicated findings. Eight studies specified ethical approval details [45–48, 50, 52, 54, 59]. Fifteen studies reported their sampling strategies, with purposeful sampling used in five [41, 49, 52–54], snowball sampling in three [54, 56, 59] and one each for multi-stage [58], convenience [48] and maximum variation sampling [50]. Twelve studies declared no conflicts of interest [41, 42, 45–47, 49, 50, 52–54, 58, 59].

### Themes

A total of 116 themes were extracted from 19 papers as depicted in Table 2 below. Themes were noted from each study through iterative reading and examining the study backgrounds. Most studies explicitly defined themes in the results, while others listed them under categories like barriers and facilitators. These themes were reduced by repeated reading, rigorous comparison and extraction of keywords and metaphors, then condensed into a final dataset for reciprocal translation [40]. Given the relatively small number of studies, a theme list table was preferred over using qualitative software like NVivo, which suits larger concept sets [36].

**Table 2:** List of included studies and themes.

No	Study Title	Major Findings	Themes
1	Dismantling Socio-Cultural Barriers to Eye Care with Tele-Ophthalmology: Lessons from Alberta Cree Community [55]	Incorporating cultural rituals and recruiting local nurses increased Aboriginal community participation.	• Cultural Context
2	Clinicians’ Perspectives on Using Mobile Eye Fundus Cameras to Screen Diabetic Retinopathy in Primary Care [57]	Clinicians are optimistic about early DR detection; concerns about the learning curve, image quality and challenges with elderly patients with cataracts or opioid use.	• Timely detection • Integration with diabetes management • Coverage increases • Device operation • Identifying users • Difficult with elderly
3	Evaluation of Multi-level Barriers and Facilitators in a large diabetic retinopathy screening program [41]	Using the CFIR framework: barriers include ungradable images, low priority, lack of staff/space; facilitators include leadership engagement, champions and training.	• Patient attitude/comfort • Image gradeability • Exam length • Provider/patient priority • Referral issues • Resources/time • Report availability • Service development • Equipment space • Reminders • Workflow • Integration • Leadership • Targets • Training • Champions • Financial constraints
4	Barriers and Facilitators of Diabetic Retinopathy Screening Utilisation in a High-Risk Population [42]	Visual symptoms motivate care-seeking; low DR knowledge; facilitators: screening cost, insurance, GP referral; barriers: other health priorities.	• Current vision • Other illnesses •Transport/finance • Provider communication • Referral • DR knowledge
5	Understanding the Knowledge Gap Experienced by US Safety Net Patients in Teleretinal Screening [7]	Patients are confused about DR severity, screening benefits, staff proficiency, referral and follow-up processes, need for counselling.	• Time/financial issues • DR confusion • Screening utility • Technology anxiety • Referral confusion • Patient-provider interaction
6	What do patients with diabetes think of the Australian model of Diabetic Retinopathy screening? [53]	Patients value accessibility; providers see early detection benefits but highlight unclear follow-up responsibility and need for better GP-ophthalmologist coordination.	• Accessibility • Economic impact • Service quality/safety • Holistic diabetes care • Staff training • Operational integration • Provider communication • Result coordination • Service improvement
7	Perceived Barriers to Diabetic Eye Care [43]	Financial issues major patient barrier; lack of DR understanding; poor GP-ophthalmologist coordination due to documentation and staff turnover.	• Financial aspects • Coordination • DR knowledge • Appointment ease • Priority
8	Determinants of Implementation of AI-supported device for DR screening [58]	Providers distrust AI grading due to liability fears; need rigorous training; call for user-friendly, interoperable tech integration.	• Screening attitude • Organisational culture • Screening time • Financial aspects •Education/training • Ophthalmologist support • Technical integration • Professional impact • Patient benefit • Data security
9	Factors Affecting Compliance with Diabetic Retinopathy Screening: English vs Spanish Speakers [49]	Non-English speakers show more confusion about DR progression, instructions and appointments.	• DR knowledge • Provider communication • Screening priority • Community support • Financial factors
10	Primary Care Provider Views of Current Referral-to-Eye Process [44]	Poor specialist coordination due to a lack of EMR referral notes or paper-based systems; GPs rely on patients to arrange appointments and seek feedback.	• Specialist feedback • Economic factors • Referral difficulty • Patient reliance • Paper referral inefficiencies
11	Patient and Provider Perspectives on barriers to screening in southern India [50]	Patients manage diabetes well but lack DR awareness due to the asymptomatic nature; labourers struggle to attend screening; providers note awareness gap.	• Diabetes impact on QoL • Eye care practices • DR knowledge • Travel/testing costs
12	Factors influencing patient adherence in rural communities [45]	Screening cost and insurance concerns; insufficient provider information on the diabetes-blindness link; call for better provider advice.	•Personal/community factors • Geographical barriers
13	Barriers, facilitators and strategies to increase teleophthalmology use in rural US clinics [46]	Patient barriers: poor DR understanding; specialist recommendations improve uptake; provider barriers: poor digital record interoperability, no appointment reminders.	• Patient knowledge • Communication • GP-endocrinologist coordination • Operational challenges
14	Planning diabetic retinopathy services in Sub-Saharan Africa [59]	Lack of up-to-date patient info and referral workflows; need better info sharing, task-sharing and addressing patient misconceptions; long waits discourage attendance.	•Policy/governance • Provider integration • Referral mechanisms • Workforce training •Financial/technical challenges
15	Implementation of Teleophthalmology to Improve DR Surveillance [47]	Fundus camera seen as innovative; workforce and ‘super-user’ champions critical; debate on modifying or redesigning protocols.	• Patient convenience • Staff training/engagement • Champions • Referral/follow-up mechanisms • Financial issues
16	Experiences of Patients with Diabetic Retinopathy [52]	Limited DR knowledge: Many patients present late with complications; poor provider guidance leads to neglect.	• Knowledge gaps • Lifestyle adherence • Community support • Physician interaction • Education
17	Stakeholder perceptions affecting teleophthalmology implementation [48]	Telemedicine benefits remote patient comfort and resource use; it needs clear guidelines and staff training.	• Screening advantages • Infrastructure facilitators •Training/protocols
18	Barriers and facilitators to DR screening in Australian primary care [54]	High equipment costs threaten program viability; GPs limit roles to referrals; educators/nurses as ‘super-users’ are suggested.	• Physician awareness • GP role • Camera operation concerns • Staff training time • Financial issues • Champions needed
19	Perceptions of Teleophthalmology Screening in Urban Primary Care [56]	Patients are satisfied and adhere to referrals despite low DR knowledge; providers are divided on telemedicine value in urban vs rural; call for integrated screening/referral in primary care.	• Patient-centred teleophthalmology • Primary care access • Patient knowledge gaps • Provider interest varied • Need for streamlined services

After noting down the themes identified by authors, each theme in each study was compared with other themes in an iterative manner to check for similarity, and therefore the common themes were grouped together into the relevant categories to create new higher-order themes.

As depicted in the above Table 3, five higher-order themes emerged from the studies: lack of knowledge, economic factors, provider challenges, ease of integration and screening benefits, all closely linked to Social Cognitive Theory [61]. This theory explains how human behaviour in social contexts is influenced by others to gain psychological safety [61]. Patients often avoid screening due to low awareness or the asymptomatic nature of diabetic retinopathy, a behaviour spreading contagiously. Consequently, low demand leads providers to deprioritize screening, hindered by financial and operational challenges.

**Table 3:** Summary of themes derived from meta-ethnography.

**Similar Concept Groups (Second-Order Themes)**	**First-Order Data (Verbatim Quotes)**	**Second-Order Data (Original Themes Identified by Authors)**
**Patient Attitude**	‘At the beginning, I couldn’t see clearly, but I didn’t pay attention... doctor told me my fundus was bleeding badly...’ [52 p.5] ‘I had a question—terms like diabetic retinopathy… macular regeneration… cataracts… are those different ailments?’ [7 p.4] ‘I don’t see an advantage to yearly checks unless having issues…’ [46 p.4] ‘[The screening staff] are kids, no specialists…’ [7 p.6] ‘The nurses are all Aboriginal so we understand each other better’ [55 p.4] ‘Diabetes won’t affect me because I have lenses... why get an eye exam?’ [42 p.4]	Cultural Context [55], Patient Attitude [41], Patient Comfort [41], Length of Exam [41], Priority for Patients [41], Appointment Reminders [41], Current Vision [42], Other Health Problems [42], Communication with Providers [42], Referral from Providers [42], Knowledge about Diabetic Retinopathy [42, 43, 49, 50], Confusion about Diabetic Retinopathy [7], Utility of Screening [7], Anxiety about Technology [7], Interaction Patient-Provider [7, 46, 52], Ease of Appointments [43], Community Assistance [49, 52], Economic Factors [44], Quality of Life [50], Personal & Community Factors [45], Lack of Knowledge [46, 52, 56], Coordination of Care [46], Educational Support [52], Patient-centred Teleophthalmology [56]
**Economic Factors**	‘Insurance doesn’t cover 100%...’ [49 p.5] ‘My husband doesn’t like to drive; I do most driving, hard to get to appointments.’ [45 p.6] ‘Returns on investment are an issue if equipment just sits unused.’ [54 p.5] ‘Cancelled appointments due to new job, now rescheduling.’ [42 p.3]	Financial Constraints [41], Transport & Financial Resources [42], Time & Financial Issues [7], Economic Impact & Effectiveness [53], Financial Aspects of Screening [43, 49, 58], Expenses of Travel & Testing [50], Geographical Limitations [45], Financial Issues [47, 54, 59]
**Challenges for Providers**	‘I don’t know how to evaluate images or urgency...’ [57 p.5] ‘Despite automation, errors occur; bad pictures biggest challenge.’ [41 p.4] ‘Patients have many urgent issues, screening gets deprioritised.’ [41 p.6] ‘Gap between image taken and report returned.’ [53 p.11] ‘No easy way to check if screening done; patients don’t remember.’ [46 p.6]	Learning Device Use [57], Difficulty with Elderly [57], Image Gradeability [41], Provider Priority [41], Referral Issues [41, 44, 59], Screening Report Availability [41, 53], Staff Training & Education [41, 47, 53, 58], Communication & Coordination [43, 53, 54, 59], Attitude to Screening [58], Time & Workflow [41, 58], Impact on Practice [58], Feedback from Specialists [44], Paper-based Inefficiencies [44], GP Roles & Knowledge [54], Concerns on Fundus Camera Proficiency [54], Lack of Provider Interest in Teleophthalmology [56]
**Ease of Integration**	‘Structural changes needed, must remember to structure processes.’ [58 p.10] ‘Difficult teaching about blindness causes due to lack of resources and expertise.’ [59 p.8] ‘Clear scheduling templates and streamlined exams essential.’ [48 p.5] ‘Reports scanned in EMR, but fields not auto populated, work ongoing.’ [41 p.7] ‘Patients confuse optometrists and ophthalmologists; unaware screening is free.’ [56 p.4]	Integration with Diabetes Management [57], User Identification [57], Time & Human Resources [41], Service Development [41], Equipment Space [41], Data Workflow [41], Examination Methods [41], System Integration [41], Leadership Interaction [41], Performance Targets [41], Continuous Interaction [41], Champions & Organisational Culture [41, 47, 54, 58], Inclusion in Holistic Care [53], Operational & Technical Challenges [46, 58, 59], Policy & Governance [59], Referral Mechanisms [47], Infrastructure Facilitators [48], Access at Primary Care [56], Provider Perception & Streamlining Need [56]
**Benefits of Screening**	‘Early diagnosis possible if screening habitual; not just when complaints arise.’ [57 p.4] ‘Patients love community screening; zero wait times; appreciate local service.’ [48 p.3] ‘Without this service, many in communities wouldn’t get reviewed due to lack of means.’ [53 p.4]	Timely Detection [57], Increased Testing Coverage [57], Greater Accessibility [53], Quality & Patient Safety [53], Patient Benefits [58], Increased Convenience [47], Screening Advantages [48]

### Patient attitude

From patients’ perspectives, attitude towards Diabetic Retinopathy (DR) is the most important factor linked to poor attendance at screening camps and preventative eye care [7, 42, 43, 46, 47, 50, 52, 56]. Many patients are unaware of diabetes’ vision-threatening effects or underestimate its dangers due to its asymptomatic nature, leading to low perceived risk and priority [49]. A US study found patients feared blindness but did not understand the need for annual screening, and absence of symptoms discouraged eye care [43]. Providers note patients lack education on diabetes and DR, highlighting the need for community outreach via posters, pamphlets and Diabetes Educators travelling with screening teams [47, 53]. Poor DR awareness exists even among some General Practitioners and community health workers, as observed in studies from the USA, Australia and Germany, contributing to inadequate counselling and patients relying on family or friends for information [42, 53, 58]. In China, patients misattributed visual symptoms to myopia, delaying intervention [52]. Proactive EHR reminders and primary care screening have proven effective [45, 46, 56].

### Economic factors

Financial reasons were a prominent theme from both patient and provider perspectives regarding the lack of diabetic retinopathy screening. Patients cited screening costs, travel expenses and income loss due to work absence, especially in the USA, where fragmented healthcare limits insurance coverage [42, 45, 46, 49, 53]. One US study found financial stress from managing Diabetes, like insulin costs, outweighed eye care concerns without symptoms [43]. Providers focused on infrastructure expenses, such as fundus cameras, internet, software and training, viewing low patient demand as limiting cost-effectiveness. These findings were reportedly primarily in studies from the USA, Australia and Germany [41, 54, 58]. Educating patients and government support for integration are essential for long-term savings [47, 57].

### Challenges for providers

Challenges for providers were a prominent theme, mainly technical issues like training, screening time, image gradeability, proficiency concerns and role confusion over referral and follow-up [7, 41, 43, 44, 48, 53, 54, 57–59]. The EyeFundusScope A008 camera’s large size required fixed calibration, limiting mobility across departments [57]. Lack of training reduced confidence, especially for elderly patients with cataracts or constricted pupils [41, 47, 48, 53, 54]. General Physicians faced uncertainty in assessing image quality or abnormalities [57]. Australia suggested an Ophthalmologist ‘buddy’ for guidance [53], but a lack of software to track referrals hindered coordination [41, 44, 47, 53, 56, 59], causing missed appointments and harm. Proper referral mechanisms and role clarity are needed.

### Ease of integration

Earlier in the ‘Patient attitude’ section, patients showed low priority for diabetic retinopathy, echoed by providers facing implementation challenges such as logistics, data security and cultural changes [45]. A key issue was integrating screening reports into existing EHR workflows without disrupting services [41]. It is advised to have Ophthalmologists, Medical Administrators and technical stakeholders on-site during the initial launch, alongside clear emergency guidelines to manage both potential breaches in data privacy and safety hazards within the work environment [48]. Simple, intuitive interfaces compatible with multiple platforms are vital to avoid vendor lock-in and ease fundus image transfer [58]. Leadership engagement and ‘Champions’, often nurses, are essential for program accountability and staff training [41, 47, 54]. AI use excited many, though legal liability concerns call for strong judicial frameworks [48, 58].

### Benefits of screening

The benefits of screening were widely agreed upon by providers, particularly in studies from Australia, Portugal and Canada where early detection and improved access were emphasized [53, 56, 57]. Many valued the potential for early detection of diabetic retinopathy to prevent severe vision loss, advocating opportunistic screening within diabetes management programmes to prioritize urgent cases [57]. The portability of mobile screening cameras was seen as expanding patient reach [53, 57] and improving patient comfort by reducing travel to distant Ophthalmologists [47, 48]. Improved access was crucial for financially constrained patients unable to take work leave [53], a view echoed in the ‘Patient Attitude’ section. Providers supported more frequent visits to rural centres, though integration methods remain debated [53].

## DISCUSSION

The literature review for this paper established that patient non-attendance at Diabetic Retinopathy screening camps largely depends on two factors: lack of knowledge about the disease and its serious consequences and distrust in the technology’s effectiveness [31, 62, 63]. This paper’s findings support these earlier studies, indicating that the asymptomatic nature of Diabetes contributes to patients’ nonchalance, as they believe no treatment is needed without visual symptoms. Awareness levels vary by setting; studies in Saudi Arabia [6, 28, 64] showed better awareness of Diabetes’ vision-threatening effects compared to those in India [5, 65–67], likely linked to economic development. For instance, Kerala, India’s most literate state, demonstrated high awareness and better prevention measures [68]. Additionally, few patients (16–36%) receive screening information from physicians; most rely on mass media such as newspapers (26.2%) and television (85.2%) [69]. Hence, educational programmes providing counselling could significantly increase screening attendance.

Financial constraints were also prominent, particularly in US studies due to healthcare fragmentation across states [4, 70, 71], a theme echoed here as half the papers were US-based. Loss of income from attending during work hours discouraged attendance globally, suggesting insurance-linked employment systems could allow paid leave for screening. Provider-side limitations reflected literature findings on training, image gradeability and referral to Ophthalmologists [4, 72, 73]. Image gradeability may improve with mydriasis, though this paper found limited support [74–77]. Inefficient referral pathways, linked to poor coordination between General Physicians and Ophthalmologists, was another barrier, mirroring the Shanghai Diabetic Eye Study where only 50% of screen-positive patients pursued treatment [78].

‘Task-sharing’, involving community health workers for education and fundus camera operation, was supported mainly in developing countries [51, 60, 76, 79, 80] but was limited here due to the developed-country focus. One African study endorsed task-shifting through Ophthalmic nurse training, but regulatory approval is pending [59]. Conversely, a US study using medical assistants to capture images revealed patient insecurity, perceiving operators as underqualified ‘kids’ [7], aligning with literature showing patient preference for specialists [81]. This hesitance affects acceptance of Telemedicine, especially with frequent visits [82]. Tailoring screening camps to local culture improves uptake; e.g. a Canadian study with Aboriginal patients increased attendance by 65% via Aboriginal nurses and traditional rituals involving spiritual leaders [55].

### Limitations

This systematic review was done by a single researcher as opposed to a team of researchers to check whether the analysis was done properly according to the transcripts and validate the findings.

## CONCLUSION

The evidence in this paper aligns with prior studies. Patient themes include lack of knowledge, awareness and financial constraints. Provider views highlight inadequate training and unclear referral pathways, causing follow-up confusion. These issues lead to low prioritisation of Diabetic Retinopathy screening by both groups. The ‘Ease of Integration’ theme suggests appointing ‘Champion’ users, using open-source technology and interoperable data, requiring strong leadership with clear goals and reviews. Evidence from four African countries shows specialist-led policy is crucial, as screening arose from severe, undiagnosed cases. This study confirms the problem’s scale and screening effectiveness for public health.

## Data Availability

The data underlying this article are derived from previously published studies, which are cited in the reference list. No new primary data were generated or analysed for this systematic review. Extracted data supporting the findings of this study are available from the corresponding author upon reasonable request.
